# Multicenter performance evaluation of the “quanty TOXO (RH region)” kit (Clonit) for molecular diagnosis of toxoplasmosis

**DOI:** 10.1128/jcm.00538-25

**Published:** 2025-10-09

**Authors:** Céline Nourrisson, Emmanuelle Varlet, Juliette Guitard, Hélène Guegan, Cécile Nabet, Jean Menotti, Hervé Pelloux, Marie-Pierre Brenier-Pinchart, Yvon Sterkers

**Affiliations:** 1 Parasitology-Mycology Laboratory, 3IHP, University Hospital Center of Clermont-Ferrand 55174 , Clermont-Ferrand, France; 2 Molecular Biology group of the French National Reference Center for Toxoplasmosis, Montpellier, France; 3 Department of Parasitology-Mycology, University of Montpellier, CNRS, IRD, University Hospital Center of Montpellier, MiVEGEC 27037, Montpellier, France; 4 Parasitology-Mycology Laboratory, Saint-Antoine Hospital, Assistance Publique-Hôpitaux de Paris 26930, Paris, France; 5 Centre de Recherche Saint-Antoine, INSERM 27102, Paris, France; 6 Sorbonne University 27063, Paris, France; 7 Parasitology-Mycology Laboratory, University Hospital Center of Rennes, Rennes, France; 8 Service de Parasitologie-Mycologie, Groupe Hospitalier Pitié-Salpêtrière, Assistance Publique–Hôpitaux de Paris 26930, Paris, France; 9 Institut Pierre-Louis d’Épidémiologie et de Santé Publique (IPLESP), Inserm 27102, Paris, France; 10 Parasitology-Mycology Laboratory, University of Lyon 133614 , Lyon, France; 11 Parasitology-Mycology Laboratory, University Hospital Center of Grenoble Alpes, Grenoble Alpes University, Grenoble, France; Mayo Clinic Minnesota, Rochester, Minnesota, USA

**Keywords:** *Toxoplasma gondii*, toxoplasmosis, molecular diagnosis

## Abstract

The diagnosis of congenital toxoplasmosis or disseminated toxoplasmosis in immunocompromised patients nowadays relies on molecular tools, in particular real-time PCR. There are many reagents on the market, and their evaluation by independent experts provides valuable information to medical biologists who are looking for a high-performance kit among the different references. Under the aegis of the French National Reference Center for Toxoplasmosis, we report here a multicenter evaluation of the analytical and clinical performances of the “quanty TOXO (RH region)” PCR assay manufactured by Clonit. The kit showed good analytical performance, as indicated by the results of serial dilution tests and external quality control samples. PCR efficiencies varied from 95% to 105%; linearity zone extended over four log units (*R*² >0.99), and limit of detection varied from <1 parasite/mL to between 1 and 5 parasites/mL, i.e., from <0.08 parasite/PCR to between 0.2 and 1 parasite/PCR, depending on the center. Based on 141 cryopreserved DNAs from a large range of clinical specimens, we determined a clinical sensitivity of 94.7% (71/75; 95% confidence interval [CI]: 87.1%–97.9%) and a clinical specificity of 100% (66/66; 95% CI: 94.5%–100%). Four false negative results were detected despite amplification carried out in duplicate. Overall, the “quanty TOXO (RH region)” PCR assay demonstrated satisfactory analytical and clinical performances for the diagnosis of toxoplasmosis, even using extraction and amplification techniques or biological matrices not validated by the manufacturer.

**IMPORTANCE:**

Due to its speed and accuracy, PCR is now the gold standard for diagnosing congenital and disseminated toxoplasmosis. High-performance molecular testing is essential, especially for immunocompromised patients and congenital infections, to initiate early treatment. This diagnostic approach increasingly relies on commercial assays. However, commercially available kits do not guarantee performance. In this study, conducted by the French National Reference Center for Toxoplasmosis, we performed an independent multicenter evaluation of the “quanty TOXO (RH region)” PCR assay manufactured by Clonit. Our results showed that this kit delivered satisfactory results for routine diagnostic use. However, among the 141 clinical samples tested, four false negative results were noted, corresponding to specimens with low parasitic load.

## INTRODUCTION

*Toxoplasma gondii* is an obligate intracellular parasite belonging to the phylum of *Apicomplexa*. This globally distributed protozoan is capable of infecting a wide range of hosts, from mammals to birds, causing toxoplasmosis (1). In humans, the most serious manifestations result in localized or disseminated damage during congenital toxoplasmosis and a life-threatening disease in immunocompromised patients. Nowadays, the diagnosis relies on molecular methods, in particular, real-time PCR. Commercial kits are becoming increasingly popular due to their ease of use and compliance with accreditation standards compared to the in-house developed methods. However, the marketing of a kit in itself is not always a guarantee of satisfactory performance (2). Indeed, even if the manufacturers report a detection threshold, the performance of the assays may vary depending on the type of sample tested or the method used to extract DNA or amplify the target DNA. The French National Reference Center for Toxoplasmosis (NRC-T), as part of one of its expertise missions, regularly evaluates new commercial kits (3–8). In 2012, it previously demonstrated the poor performance of the ready-to-use nested-PCR AMS94/F assay (Clonit, Milan, Italy) (2). Indeed, the evaluation concluded that the method lacked sensitivity and detected only 50% of positive amniotic fluids. Nowadays, Clonit markets a new assay called “quanty TOXO (RH region)”. In the present study, the analytical and clinical performance of this new kit was evaluated using a multicenter strategy allowing for testing various extraction and amplification platforms currently widely used in medical diagnostic laboratories on a large range of matrices.

## MATERIALS AND METHODS

### Study design

This multicenter study involved seven medical Parasitology-Mycology laboratories from French university hospitals, experts in the diagnosis of toxoplasmosis, all belonging to the working group of the Molecular Biology Pole of the French NRC-T (https://cnrtoxoplasmose.chu-reims.fr/?lang=en, last accessed 1 April 2025). All participating centers are accredited (i) according to the ISO 15189 norm for the molecular diagnosis of toxoplasmosis and (ii) by the French Ministry of Health (Regional Health Agency) for the prenatal diagnosis of congenital toxoplasmosis.

In the first part of the study, two of the seven centers (centers 1 and 2) evaluated (i) the analytical sensitivity and PCR efficiency of the “quanty TOXO (RH region)” PCR assay using a serial dilution from a calibrated *T. gondii* lyophilized standard or using a patient specimen matrix spiked with lyophilized *T. gondii* (see below) (9) and (ii) the technical agreement between their reference PCRs and the “quanty TOXO (RH region)” PCR assay using samples from external quality assessment (EQA) programs (see below).

In the second part of the study, all the seven centers (centers 1 to 7) included clinical specimens from hospital collections to evaluate clinical performance of the “quanty TOXO (RH region)” PCR assay.

### Samples

Freeze-dried calibrated suspensions (10^4^ *Toxoplasma*/mL, *T. gondii* type II) (10) were extracted (i) manually by center 1 using an alkaline thermolysis from 200 µL of the pellet with TNN lysis buffer (0.5% Tween-20, 0.5% Nonidet P40, 10 mM NaOH) as described elsewhere (11) and (ii) with an automated platform by center 2 with ELITe InGenius SP 200 cartridges (ELITechGroup, Inc., Bothell, WA) (nucleic acids from 200 µL of the pellet) on the ELITe InGenius platform (ELITechGroup, Inc.), as previously described (8). In both extractions, the elution volume to recover the DNA was 100 µL and no internal control (IC) was added. Extracted DNA was then 10-fold diluted in both centers to obtain a range of concentrations from 10,000 to 0.01 T. *gondii* genome/mL. Then in both centers with their own DNA extract, the highest concentration at 10,000 T. *gondii* genome/mL was tested in duplicate, the three following concentrations at 1,000, 100, and 10 T. *gondii* genome/mL were tested in triplicate, and the three lowest concentrations, until 0.01 T. *gondii* genome/mL, were analyzed in six wells each.

To determine the limit of detection of the PCR assays, *Toxoplasma gondii*-negative amniotic fluids were spiked with freeze-dried calibrated suspensions of 10 *Toxoplasma*/mL in order to obtain specimens containing 10, 5, and 1 T. *gondii* genome/mL. These three specimens were extracted (i) manually by center 1 using an alkaline thermolysis from 2,000 µL with TNN lysis buffer with an elution volume of 250 µL and (ii) with an automated platform by center 2 with ELITe InGenius SP 1000 cartridges (ELITechGroup, Inc., Bothell, WA) (nucleic acids from 1,000 µL) on the ELITe InGenius platform with an elution volume of 100 µL, as previously described (8). Each extract was tested 20-fold.

A panel of Quality Control for Molecular Diagnostics (Glasgow, Scotland, UK) quality controls was also analyzed by these two centers. It consisted of four vials of freeze-dried amniotic fluid samples and one vial of freeze-dried plasma samples spiked or not with various concentrations of *T. gondii* (TGDNA22C2 panel). All these freeze-dried samples were reconstituted by adding 2.0 mL of sterile molecular-grade water, as recommended by the manufacturer. Suspensions were centrifuged after 5 minutes, then supernatant was discarded, and DNA from the pellet (200 µL or 1,000 µL, see below) was extracted. Center 1 carried out a manual DNA extraction for the serial dilution assay (11). The “quanty TOXO (RH region)” IC was added either before the extraction or afterward directly into the PCR mix (elution 100 µL) to check the absence of competitive effect induced by the IC included in this PCR assay. Center 2 carried out an automated DNA extraction without adding IC, using the extraction cartridges ELITe InGenius SP 1000 (nucleic acids from 1,000 µL of the pellet eluted in 100 µL) on the ELITe InGenius platform. Each extract was tested in duplicate.

The clinical samples included in the second part of the study were DNA previously extracted in each of the seven centers as part of routine diagnosis (see Table 1 for extraction methods of each center) and stored at −20°C for 6 years for the oldest one. Each center was asked to suggest 10 positive and 10 negative DNAs by matching the initial biological matrix in each of the groups (e.g., one *T. gondii*-positive and one *T. gondii*-negative placenta), verifying that there was sufficient volume for the entire study. The samples were classified as positive or negative according to the reference PCR result of each laboratory (see Table 1 for amplification methods of each center). Before inclusion in the study, patients and samples were carefully categorized according to the definitions proposed (i) by Lebech et al. for congenital toxoplasmosis, with comparative immunoblot (IgG and IgM) at birth performed to support diagnosis (12), (ii) by Martino et al. for infections in immunocompromised patients (13, 14), and (iii) by Fekkar et al. for ocular toxoplasmosis (15). Each extract was tested in duplicate.

**TABLE 1:** Reference methods used in each center for the routine diagnosis of toxoplasmosis and PCR platforms tested for Clonit PCR assay

Center	Extraction	Reference method		“quanty TOXO (RH region)”
		PCR assay^*a*^	PCR platform	PCR platform
1	TNN^*b*^ (11) or Protein Precipitation (Promega) (16)	Reischl et al. (17)	LC480 (Roche)	LC480 (Roche)
2	ELITe InGenius (ELITech)	*Toxoplasma* ELITe MGB assay (ELITech) (8)	ELITe InGenius (ELITech)	Rotor-Gene Q (Qiagen)
3	QIAamp DNA Mini Kit (Qiagen)	Reischl et al. (17)	LC480 (Roche)	LC480 (Roche)
4	QIAsymphony (Qiagen)	Bio-Evolution *Toxoplasma gondii* detection kit (5)	LC480 (Roche)	QuantiStudio 5 (Applied Biosystems)
5	QIAamp DNA Mini Kit or EZ1 Advanced XL (Qiagen)	Robert-Gangneux et al. (18)	QuantiStudio 5 (Applied Biosystems)	QuantiStudio 5 (Applied Biosystems)
6	Emag (bioMérieux) or QIAamp DNA Mini Kit (Qiagen)	Fekkar et al. (15)	QuantiStudio 5 (Applied Biosystems)	QuantiStudio 5 (Applied Biosystems)
7	MagNA Pure 96, MagNA Pure Compact (Roche)	Bio-Evolution *Toxoplasma gondii* detection kit (5)	QuantiStudio 5 (Applied Biosystems)	QuantiStudio 5 (Applied Biosystems)

^*a*^ All PCR assays target rep529 (19).

^*b*^ TNN: alkaline thermolysis (Tween-Nonidet-NaOH) was used for paucicellular fluids and the Protein Precipitation protocol (Promega) for tissues and cellular samples.

### “quanty TOXO (RH region)” PCR assay (Clonit)

The “quanty TOXO (RH region)” kit (ref: RT-94, Clonit) was validated by the manufacturer to be used with extracted DNA from whole blood collected in EDTA or amniotic fluid. We used the kit according to the leaflet’s instructions except for the IC. Indeed, the IC must be added prior to extraction, but as the second part of this study is based on previously extracted DNA, we had to determine the amount of IC to add to the PCR mixture to get as close as possible to the kit's usual conditions of use. Our preliminary tests showed that it was necessary to add 0.4 µL of IC prediluted 1/61 into the reaction tube with 20 µL of master mix and 5 µL of eluted DNA in order to obtain a Ct around 26 (data not shown). The set parameters for DNA amplification complied with the protocols defined by each manufacturer. Samples with Ct <45 were considered positive. Clinical and control samples were tested in duplicate. In case of inhibition of amplification (Ct of IC >28), DNA extract was diluted 1/10 in sterile water, and the dilution was tested in duplicate. In case of discrepant qualitative results between reference PCR and “quanty TOXO (RH region)” PCR, samples were re-analyzed in duplicate in both qPCR assays (to ensure that the DNA extract was well preserved).

Cross-reactivity was assessed by center 2 using DNA extracts positive for different pathogens (see the list in the result section) that could be detected in blood and which were obtained as part of routine laboratory activity. Each DNA extract was tested in duplicate.

### Statistical analysis

Crossing threshold (Ct) values were used to elaborate the standard curve and to determine the efficiency of each PCR method. ΔCt is the difference between Ct values obtained with each assay for the same sample. Means, SDs, coefficients of variation (CVs), and correlations (*R*²) were calculated using Microsoft Excel version 16.0.5474.1000 software (Microsoft, Redmond, WA, USA). In the first part of the study, slopes of standard curves allowed determination of PCR efficiency in conformity with the formula [10^(-1/slope)^ − 1] * 100. Slopes were calculated when all replicates were positive. Slopes between −3.1 and −3.6 giving reaction efficiencies between 90% and 110% are typically acceptable (20). Linearity and range were evaluated by constructing a calibration curve when all multiplicates were positive. Limit of detection (LOD) was evaluated with spiked amniotic fluids and corresponded to the quantity of *T. gondii* for which the test was positive 95% of the time. In the second part of the study, clinical performances were assessed by comparing qualitative results and quantitative Ct values obtained for clinical specimens between the commercial kit and the reference PCR of each laboratory. Qualitative results of commercial PCR were (i) expressed as performance scores, corresponding to the number of concordant PCR out of the total number of PCR reactions performed, and (ii) analyzed through sensitivity and specificity calculations obtained after the first run (and DNA extract dilution in case of absence of IC amplification); the gold standard was the final diagnosis. Quantitative Ct values were compared by performing Bland-Altman plots (Microsoft Excel), which is a graphical representation of the difference of Ct in the abscissa plotted against the mean of Ct values in the ordinate, for each sample analyzed by both assays (21).

## RESULTS

### PCR efficiency, performances, and limit of detection using *Toxoplasma*-calibrated suspensions and spiked samples

Serial dilutions of a calibrated *Toxoplasma* suspension were performed from 10,000 to 0.01 parasites/mL by centers 1 and 2 with their own reference PCR and the Clonit kit. Qualitative and quantitative results are reported in Table 2. All PCR efficiencies were between 95% and 107%, and linearity on five 10-fold dilutions had *R*² >0.99. In terms of sensitivity, the lowest parasite concentration (i.e., 0.01 *Toxoplasma*/mL) was inconsistently amplified, in all conditions, regardless of the extraction or PCR strategy among those tested. The concentration of 0.1 parasite/mL was inconsistently amplified with “quanty TOXO (RH region)” PCR assay, only when DNA was extracted with ELITe InGenius platform (see center 2 results on Table 2). So, the PCR performance score was the lowest in this condition (65.5%) compared to all other conditions where it exceeded 80%. The LOD of the Clonit assay was less than 1 parasite/mL with extraction and amplification combination of center 1 (compared to 5 parasites/mL with its reference method), and was between 1 and 5 parasites/mL for center 2 (compared to <1 parasite/mL with its reference method) (Table 3; Table S1). Taking into account, on the one hand, the sample volume for DNA extraction and the elution volumes, and on the other hand, the input volumes for PCR assays (10 µL for Clonit assay, 5 µL for center 1 reference PCR and 10 µL for center 2 reference PCR), the LOD of Clonit assay can also be expressed as less than 0.08 parasite/PCR for center 1 (and 0.2 parasite/PCR with its reference method), and between 0.2 and 1 parasite/PCR for center 2 (compared to <0.2 parasite/PCR with its reference method).

**TABLE 2:** Performance of the “quanty TOXO (RH region)” PCR assay using DNA serial dilutions of a calibrated *Toxoplasma* suspension^*a*^

Concentration, *T. gondii* parasites/mL	Center 1		Center 2	
	Reference PCR	Clonit PCR	Reference PCR	Clonit PCR
10,000	2/2	2/2	2/2	2/2
	20.0 ± 0.15	19.7 ± 0.01	21.4 ± 0.02	20.0 ± 0.08
1,000	3/3	3/3	3/3	3/3
	23.5 ± 0.02	23.0 ± 0.08	25.0 ± 0.06	23.5 ± 0.09
100	3/3	3/3	3/3	3/3
	26.7 ± 0.16	26.2 ± 0.26	28.6 ± 0.36	26.8 ± 0.29
10	3/3	3/3	3/3	3/3
	30.2 ± 0.45	29.3 ± 0.97	32.6 ± 0.34	29.6 ± 0.47
1	6/6	6/6	6/6	6/6
	33.5 ± 1.13	32.6 ± 0.90	34.3 ± 0.72	34.1 ± 1.43
0.1	6/6	6/6	6/6	2/6
	35.5 ± 1.32	33.5 ± 1.84	37.2 ± 0.73	35.5 ± 3.33
0.01	2/6	2/6	1/6	0/6
	34.8 ± 0.95	36.5 ± 2.93	39.5	NA^*b*^
Performance score total, (%)	25/29 (86.2)	25/29 (86.2)	24/29 (82.8)	19/29 (65.5)
Slope	−3.4	−3.2	−3.2	−3.4
Efficiency [−1 + 10^(-1/slope)^], %	98	105	107	95
*R*²	>0.99	>0.99	>0.99	>0.99

^*a*^ Qualitative results (number of positive results out of the number of wells) (top line) and Ct values (bottom line) are presented for each concentration. SDs were calculated on the basis of the positive values only.

^*b*^ NA, not available.

**TABLE 3:** LOD determination^*a*^

Concentration, *T. gondii* parasites/mL	Center 1		Center 2	
	Reference PCR	Clonit PCR	Reference PCR	Clonit PCR
10	20/20 (100)	n.t.^*b*^	20/20 (100)	20/20 (100)
	29.4 ± 0.42		31.4 ± 0.52	30.2 ± 0.35
5	19/20 (95)	20/20 (100)	20/20 (100)	20/20 (100)
	30.4 ± 0.45	33.0 ± 0.60	32.0 ± 0.39	30.9 ± 0.52
1	17/20 (85)	20/20 (100)	20/20 (100)	18/20 (90)
	37.4 ± 2.08	38.2 ± 0.89	36.5 ± 0.77	36.4 ± 1.47

^*a*^ Qualitative results (number of positive results out of the number of wells, %) (top line) and Ct values (bottom line) are presented for each concentration. SDs were calculated on the basis of the positive values only.

^*b*^ n.t., not tested.

Concerning the five EQA samples analyzed by centers 1 and 2, all results were concordant (Table S2). Of note, this type of sample enabled center 1 to test the IC provided by the kit, either by adding it upstream of the extraction as recommended by the manufacturer or by adding it directly in the PCR mix with the extracted DNA. Regarding the four positive samples, all qualitative results were concordant regardless of the extraction technique or when the IC was added. Regarding the quantitative results of the positive samples obtained with the “quanty TOXO (RH region)” PCR assay with and without IC obtained by center 1, Ct values showed good agreement. Thus, for the TGDNA22C2-02 sample (i.e., 32 ± 13 *T*. *gondii*/mL), ΔCt was 0.5; and for the three other samples (TGDNA22C2-03, TGDNA22C2-04, TGDNA22C2-05; i.e., 4 ± 3 *T*. *gondii*/mL), which were in fact the three same samples prepared by the manufacturer, ΔCt ranged from 0.1 to 0.7. Regarding the negative EQA samples, IC values greater than 28 were observed by center 1 regardless of whether IC was added before or after extraction. The inhibition disappeared after dilution 1/10 (Table S2).

### Clinical specimens

Among the 141 clinical specimens tested, (i) 75 were positive for *T. gondii* with the reference PCRs and corresponded to congenital toxoplasmosis (*n* = 17), cerebral toxoplasmosis (*n* = 17), probable *Toxoplasma* disease (*n* = 21), possible *Toxoplasma* disease (*n* = 1), ocular toxoplasmosis (*n* = 10), *Toxoplasma* infection (*n* = 4), pulmonary toxoplasmosis (*n* = 2), and definite *Toxoplasma* disease (*n* = 3), and (ii) 66 were sampled from patients without any *T. gondii* infection (Table S2). Eleven distinct biological matrixes were included (63 peripheral blood, 21 cerebrospinal fluids [CSF], 15 aqueous humor, 13 placenta, 11 amniotic fluids, 4 vitreous humor, 4 cerebral biopsy, 3 bone marrow, 2 cord blood, 3 bronchoalveolar lavage fluids [BALF], 2 sputum) (Table 4).

**TABLE 4:** Summary of results obtained with “quanty TOXO (RH region)” PCR assay for the 141 clinical samples

Samples (no. included in the study)	Qualitative results	No. of samples^*a*^	First duplicate			First and second duplicates when necessary		
			Concordant^*b*^	Discordant result	PCR inhibition	Concordant^*b*^	Discordant result	PCR inhibition
Peripheral blood (63)	Positive	30	26 (3)	0	1	26 (4)	0	0
	Negative	33	33	0	0			
Umbilical cord blood (2)	Positive	2	1 (0)	0	1	1 (0)	1	0
Amniotic fluid (11)	Positive	7	7 (0)	0	0	7 (0)	0	0
	Negative	4	4	0	0			
Placenta (13)	Positive	8	7 (0)	1	0	7 (0)	1	0
	Negative	5	5	0	0			
CSF (21)	Positive	12	12 (0)	0	0	12 (0)	0	0
	Negative	9	9	0	0			
Cerebral biopsy (4)	Positive	2	2 (0)	0	0	2 (0)	0	0
	Negative	2	2	0	0			
Bone marrow (3)	Positive	2	2 (0)	0	0	2 (0)	0	0
	Negative	1	1	0	0			
BALF (3)	Positive	1	1 (0)	0	0	1 (0)	0	0
	Negative	2	2	0	0			
Sputum (2)	Positive	1	1 (0)	0	0	1 (0)	0	0
	Negative	1	1	0	0			
Aqueous humor (15)	Positive	8	6 (0)	2	0	6 (1)	1	0
	Negative	7	7	0	0			
Vitreous humor (4)	Positive	2	2 (0)	0	0	2 (0)	0	0
	Negative	2	2	0	0			
Total (141)	Positive	75	67 (3)	3	2	67 (5)	3	0
	Negative	66	66	0	0			

^*a*^ Positive and negative samples according to the reference PCR and final diagnosis.

^*b*^ Results are presented as follows: number of samples with both duplicate results being concordant with qualitative reference PCR result (number of samples with different results in the duplicate, i.e., inconsistently positive results, but overall concordant with qualitative reference PCR result).

When comparing “quanty TOXO (RH region)” PCR versus reference PCRs, qualitative results of the first duplicates of PCR were concordant for 136/141 samples (96.5%). In 3/141 samples (2.1%), one placenta and two aqueous humor, results were found falsely negative with the “quanty TOXO (RH region)”. In 2/141 samples (1.4%), one cord blood and one peripheral blood were found uninterpretable due to the absence of amplification of the IC (Tables 4 and 5). These two samples were re-tested after a 1/10 dilution. Only the peripheral blood appeared positive after this dilution, whereas the cord blood remained uninterpretable. So, sensitivity was 94.7% (71/75; 95% CI: 87.1%–97.9%). The three false-negative samples obtained after the first run were re-tested in a second duplicate by the reference PCR and by the “quanty TOXO (RH region)” PCR. With the reference PCR, all three samples (but also the two samples firstly uninterpretable with the “quanty TOXO (RH region)” PCR) were positive again. With the “quanty TOXO (RH region)” PCR, only the aqueous humor was positive after the second run. In total, 3/141 samples (2.1%), one aqueous humor, one placenta, and one cord blood, remained falsely negative after the 2nd run. No false-positive result was found with “quanty TOXO (RH region)” PCR assay, so specificity was 100% (66/66; 95% CI: 94.5%–100.0%). In addition, no cross-reactivity was observed with a panel of DNAs from pathogens commonly found in clinical routine; indeed, no amplification was obtained with “quanty TOXO (RH region)” PCR assay from DNA extracts of *Plasmodium falciparum*, *Plasmodium ovale*, *Babesia venatorum*, *Leishmania infantum*, *Loa Loa*, *Mansonella perstans*, *Aspergillus fumigatus*, *Lichtheimia corymbifera*, *Escherichia coli*, *Pseudomonas aeruginosa*, cytomegalovirus, and Epstein-Barr virus.

**TABLE 5:** Focus on inconsistently positive and discordant samples^*a*^

Center	Sample	Reference PCR Ct value of *T. gondii* target					“quanty TOXO (RH region)” PCR Ct value of *T. gondii* target (Ct value of IC target)					Interpretation: after 1st run/after 1st and 2nd runs
		Qualitative result	Well 1	Well 2	Well 3	Well 4	Qualitative result	Well 1	Well 2	Well 3	Well 4	
1	Umbilical cord blood	Inc. Pos	38.7	Neg	37.2	36.3	Neg	Neg (Neg)	Neg (Neg)	Neg^*b*^ (22.5)	Neg^*b*^ (22.5)	Uninterpretable/discordant
	Peripheral blood (buffy coat)	Pos	31.2	31.1	30.0	30.0	Inc. Pos	Neg (Neg)	Neg (Neg)	35.2^*b*^ (21.8)	36.6^*b*^ (21.9)	Uninterpretable/concordant
2	Peripheral blood (buffy coat)	Pos	40.7	41.2	n.t.^*c*^	n.t.	Inc. Pos	Neg (20.7)	38.4 (20.8)	n.t.	n.t.	Concordant/concordant
	Aqueous humor	Inc. Pos	43.0	Neg	40.3	Neg	Neg	Neg (21.4)	Neg (21.4)	Neg (21.5)	Neg (21.3)	Discordant/discordant
3	Peripheral blood (buffy coat)	Pos	32.7	33.2	n.t.	n.t.	Inc. Pos	Neg (22.2)	37.4 (22.2)	n.t.	n.t.	Concordant/concordant
4	Peripheral blood (whole blood)	Inc. Pos	35.0	Neg	n.t.	n.t.	Inc. Pos	38.7 (23.3)	Neg (23.4)	n.t.	n.t.	Concordant/concordant
5	Placenta	Pos	36.0	32.85	n.t.	n.t.	Neg	Neg (18.6)	Neg (18.5)	Neg (18.5)	Neg (18.5)	Discordant/discordant
6	Aqueous humor	Inc. Pos	38.2	38.4	37.7	Neg	Inc. Pos	Neg (26.9)	Neg (26.7)	43.0 (27.2)	Neg (27.3)	Discordant/concordant
7	Peripheral blood (whole blood)	Inc. Pos	38.9	Neg	n.t.	n.t.	Pos	40.12 (22.1)	39.33 (22.0)	n.t.	n.t.	Concordant/concordant

^*a*^ Inc. Pos, inconsistently positive; Neg, negative.

^*b*^ Extracted DNA was diluted due to the absence of IC in the first run. The results obtained during the first run are indicated in columns “Well 1” and “Well 2,” and those obtained during the second run are indicated in columns “Well 3” and “Well 4”.

^*c*^ n.t., not tested.

**Fig 1:**
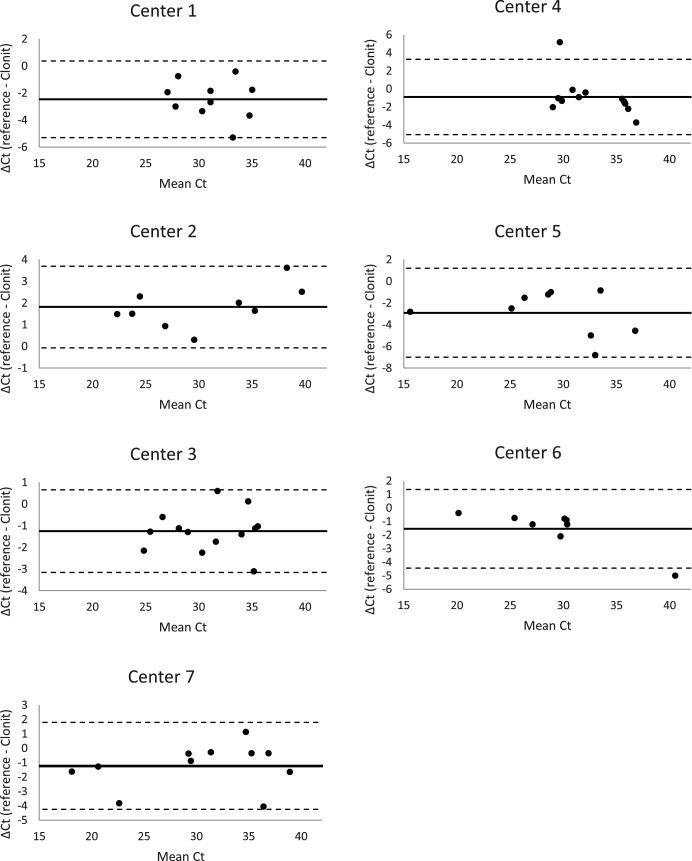
Bland-Altman plots comparing the Ct values of the reference PCR of each of the seven centers and the “quanty TOXO (RH region)” PCR for clinical specimens. For each positive sample in each center by both methods (black dots), Ct values were plotted, with the mean of both methods in abscissa and the difference between both methods in ordinate. The thick line and the dotted lines represent the mean and the ±1.96 SDs, respectively.

Concerning quantitative results, Ct values of the concordant positive samples obtained with both reference PCR in each center and “quanty TOXO (RH region)” PCR were compared and plotted in a Bland-Altman graph (Fig. 1). There was an overall good agreement of the “quanty TOXO (RH region)” PCR with the reference PCRs, with only two samples out of the range of ±1.96 SD (centers 4 and 6). The bias ranged from 0.12 to 6.79 according to the centers.

## DISCUSSION

Molecular approaches are essential for the diagnosis of congenital or disseminated toxoplasmosis, and commercial kits are now numerous on the market and widely used, at the expense of in-house assays (22). The instructions for commercial kits present performance evaluations carried out by the manufacturer. However, evaluation by independent users, under the conditions of routine diagnosis, has the advantage of using diverse samples and biological matrixes, as well as up-to-date DNA extraction techniques and amplification platforms. During these independent evaluations, it is important to test samples with low parasitic loads in order to challenge these reagents. A multicenter design of these studies involving experts in the field strengthens the results.

A first characteristic of the “quanty TOXO (RH region)” PCR assay to highlight is the choice of the *T. gondii* target, i.e., *rep529*. This repeated DNA element *rep529* (GenBank accession number AF146527) (17, 19) was shown to provide high sensitivity to qPCR assays (9, 17) and has become the main DNA target used in most PCR methods, whether in-house or commercial techniques (22). Another positive point of this reagent is that it uses uracil-DNA glycosylase, which is recommended to prevent residual contamination (22). Then, like many commercial kits, the ease of use of this reagent and the speed of preparation of its master mix are strong points. In addition to the detection of *T. gondii* genome, this kit also allows its quantification. However, the French NRC-T does not currently recommend providing quantitative results given the lack of standardization with *rep529* target (22).

To our knowledge, this is the first published independent evaluation of the “quanty TOXO (RH region)” PCR assay. In the present study, satisfactory analytical performances were obtained. The LOD, determined with two combinations of extraction/amplification methods, is good, lower than 1 parasite/PCR (or 5 parasites/mL), and slightly variable from one PCR platform to another, highlighting the importance of the extraction-amplification-reagent trio. Seven expert centers also confirmed the correct clinical performances of the “quanty TOXO (RH region)” PCR assay, with sensitivity reaching 94.7% (71/75; 95% CI: 87.1%–97.9%) and specificity of 100% (66/66; 95% CI: 94.5%–100.0%), as compared to their reference PCRs. This excellent specificity was also confirmed by the absence of cross-reactivity. Regarding sensitivity, we want to stress here that all analyses were performed in duplicate. If the analyses were performed without duplication, instead of three false negatives, we would have obtained four. Three discordant and two uninterpretable results were detected at the end of the first run in the present study, unrelated to the extraction or amplification technique used, whether validated or not by the manufacturer. After a second run of these five samples, three samples remained falsely negative. It should be noted that the three corresponding biological matrices (aqueous humor, placenta, cord blood) were not validated by the manufacturer. Concerning the retesting for discrepant samples, it is important to point out that only the two uninterpretable tests would have warranted retesting as part of a routine diagnostic procedure. That is the reason why sensitivity calculation takes into account the results obtained after dilution of these two samples. As stated above, this study also highlighted once again the benefit of carrying out amplifications in duplicate, as several samples were detected inconsistently positive by the reference PCR and by the “quanty TOXO (RH region)” PCR. Of note, two inconsistently positive samples by reference PCR were detected negative by the “quanty TOXO (RH region)” PCR even after the second duplicates. Indeed, parasitic loads are often low during disseminated or congenital toxoplasmosis, with a median concentration in amniotic fluid estimated around 10 tachyzoites per milliliter (23, 24). So, the French NRC-T recommends, in addition to the use of a highly sensitive PCR assay, the analysis of clinical samples at least in duplicate, which thus increases the probability of detecting these low loads (22). The recommendation to perform duplicate analyses should be included in the manufacturer’s documentation. It should be noted that due to certain economies of scale, the cost of the analysis will not be doubled, but when implementing a commercial technique or even an in-house method, the cost calculation must take this parameter into account. We also want to highlight that these performances tend to be lower than those obtained with the *Toxoplasma gondii* Real-TM (Sacace) or *Toxoplasma* RealCycler Universal PCR (Progenie Molecular) assays for which the NRC-T evaluations obtained clinical sensitivities and specificities of 100% (106/106; 95% CI: 96.5%–100%) and 100% (67/67; 95% CI: 94.6%–100%) or 97.8% (133/136; 95% CI: 97.8%–100%) and 100% (27/27; 95% CI: 87.2%–100%), respectively (4, 6). . However, comparisons of these values must be made with caution, as they were obtained from different clinical cohorts. It would appear that the cohort in this study contains more low-dose samples. This study included extraction techniques, PCR machines, and biological matrices not all validated by the manufacturer of the “quanty TOXO (RH region)” PCR assay. No center and/or method bias was observed in this study, demonstrating the robustness of the reagent.

The retrospective design of the present study may induce some limitations, as DNA extracts from clinical samples included have been frozen since their sampling. Yet, it has been previously demonstrated that freezing and storage at −20°C provide adequate preservation of *T. gondii* DNA for retrospective molecular analysis (25). In addition, in the present study, re-analyzing the discordant samples with the reference PCR confirmed in each case the non-degradation of the DNA. Nevertheless, it would be better to use clinical samples rather than the DNA previously extracted to more reliably assess the impact of the IC. However, this would appear to be totally impossible on a number and diversity of samples as large as those we tested, since toxoplasmosis (congenital or in immunocompromised patients) is a rare event.

Overall, the “quanty TOXO (RH region)” PCR assay offers satisfactory performance, regardless of the platform used, and constitutes a kit suitable for carrying out, in particular, systematic screening of patients after hematopoietic stem cell transplantation.

## Data Availability

Data are available in Tables S1 and S2.

## SUPPLEMENTAL MATERIAL

Supplemental legends jcm.00538-25-s0001.docx Legends for Tables S1 and S2.

Table S1 jcm.00538-25-s0002.xlsx Ct values obtained for LOD determinations.

Table S2 jcm.00538-25-s0003.xlsx Raw data.

ASM does not own the copyrights to Supplemental Material that may be linked to, or accessed through, an article. The authors have granted ASM a non-exclusive, world-wide license to publish the Supplemental Material files. Please contact the corresponding author directly for reuse.
