# Supplementary material for: Multicenter performance evaluation of the “quanty TOXO (RH region)” kit (Clonit) for molecular diagnosis of toxoplasmosis
Source: J Clin Microbiol. 2025 Oct 9;63(11):e00538-25. doi: 10.1128/jcm.00538-25 (PMC12607907; doi:10.1128/jcm.00538-25)
Supplement: Supplemental legends — Legends for Tables S1 and S2. [file jcm.00538-25-s0001.docx]

# Supplementary materials

## Supplementary Table S1. Ct values obtained for LOD determinations.

Neg: negative

## Supplementary Table S2. Raw data.

For clinical samples, in the event of a discrepancy in the qualitative result between the reference PCR and the Clonit PCR (Clonit PCR column: first duplicate, wells 1 and 2), the reference PCR and the Clonit PCR are retested in duplicate (Clonit PCR column: second duplicat, wells 3 and 4). Ct values of duplicates are shown in italics. Ct values obtained after dilution 1/10 are indicated with the symbol *.

For samples linked to (suspected) maternal infection during pregnancy, final diagnosis of congenital toxoplasmosis is adapted on case definitions according to Lebech et *al*. (13), with the only adjustment that molecular biology diagnosis is assimilated to cultivation approach. Comparative immunoblot (IgG and IgM) at birth was performed to support diagnosis (12). For blood samples in immunocompromised patients, clinical classification was either *‘Toxoplasma* disease: definite / probable / possible’ or ‘*Toxoplasma* infection’ according to the European Group for Blood and Marrow Transplantation Infectious Diseases Working Party classification (14). For cerebral / ocular / respiratory samples positive by PCR and whose clinical context or symptoms or imaging are compatible, the final diagnosis was cerebral / ocular / pulmonary toxoplasmosis (15). For peripheral blood samples, depending on centers, either buffy coat (centers 1-3 and 5) or whole blood (centers 4, 6 and 7) were extracted. BALF: bronchoalveolar lavage fluid; CSF: cerebrospinal fluid; Ct: cycle threshold; EQA: external quality assessment; IC: internal control; Inc. Pos: inconsistently positive; NA: not applicable; Neg: negative; Nb: number; Pos: positive; SD: standard deviation.
